# Radial Data Mining to Identify Density–Dose Interactions That Predict Distant Failure Following SABR

**DOI:** 10.3389/fonc.2022.838155

**Published:** 2022-03-09

**Authors:** Angela Davey, Marcel van Herk, Corinne Faivre-Finn, Alan McWilliam

**Affiliations:** ^1^ Division of Cancer Sciences, School of Medical Sciences, Faculty of Biology, Medicine and Health, The University of Manchester, Manchester, United Kingdom; ^2^ Department of Radiotherapy Related Research, The Christie National Health Service (NHS) Foundation Trust, Manchester, United Kingdom; ^3^ Department of Clinical Oncology, The Christie National Health Service (NHS) Foundation Trust, Manchester, United Kingdom

**Keywords:** image-based data mining, personalised medicine, imaging biomarkers, stereotactic ablative body radiation (SABR), biomarker-by-treatment interactions, distant metastasis, NSCLC

## Abstract

**Purpose:**

Lower dose outside the planned treatment area in lung stereotactic radiotherapy has been linked to increased risk of distant metastasis (DM) possibly due to underdosage of microscopic disease (MDE). Independently, tumour density on pretreatment computed tomography (CT) has been linked to risk of MDE. No studies have investigated the interaction between imaging biomarkers and *incidental* dose. The interaction would showcase whether the impact of dose on outcome is dependent on imaging and, hence, if imaging could inform which patients require dose escalation outside the gross tumour volume (GTV). We propose an image-based data mining methodology to investigate density–dose interactions radially from the GTV to predict DM with no *a priori* assumption on location.

**Methods:**

Dose and density were quantified in 1-mm annuli around the GTV for 199 patients with early-stage lung cancer treated with 60 Gy in 5 fractions. Each annulus was summarised by three density and three dose parameters. For parameter combinations, Cox regressions were performed including a *dose–density* interaction in independent annuli. Heatmaps were created that described improvement in DM prediction due to the interaction. Regions of significant improvement were identified and studied in overall outcome models.

**Results:**

Dose–density interactions were identified that significantly improved prediction for over 50% of bootstrap resamples. Dose and density parameters were *not* significant when the interaction was omitted. Tumour density variance and high peritumour density were associated with DM for patients with more cold spots (less than 30-Gy EQD2) and non-uniform dose about 3 cm outside of the GTV. Associations identified were independent of the mean GTV dose.

**Conclusions:**

Patients with high tumour variance and peritumour density have increased risk of DM if there is a low and non-uniform dose outside the GTV. The dose regions are independent of tumour dose, suggesting that *incidental* dose may play an important role in controlling occult disease. Understanding such interactions is key to identifying patients who will benefit from dose-escalation. The methodology presented allowed spatial dose–density interactions to be studied at the exploratory stage for the first time. This could accelerate the clinical implementation of imaging biomarkers by demonstrating the impact of *incidental* dose for tumours of varying characteristics in routine data.

## 1 Introduction

Stereotactic body radiotherapy (SABR) is standard of care for patients with early-stage non-small cell lung cancer (NSCLC) who are not eligible for surgery due to refusal or ill health (1). High dose radiation is delivered in few fractions so tight radiotherapy (RT) margins are implemented to limit the dose to surrounding normal tissues. To keep the dose conformal, the clinical target volume (CTV) is generally omitted as the dosimetric penumbra is believed to provide adequate coverage of microscopic disease extensions (MDE) that cannot be visualised on standard imaging (2, 3). Depending on the prescription dose, approximately 6 mm of MDE coverage will be provided by the dose fall-off outside the planned treatment area (4); however, it is believed that at least 2.6 cm is required for adequate coverage in 90% of patients (5). If MDE is undertreated, it can increase the risk of treatment failure (3, 6).

Following lung SABR, the predominant pattern of treatment failure is distant metastasis (DM), with a 20% failure rate in the first 5 years (7). Distant metastasis can occur due to untreated microscopic disease that spreads throughout the body *via* lymphatic, vascular, or local invasion (6, 8). The impact of inadequate coverage of MDE was demonstrated in a study where a biologically equivalent dose (EQD2) of less than 21 Gy outside the planning target volume (PTV) was associated with increased risk of DM (9). In this study, there is an implicit assumption that inadequate coverage of MDE is associated with the same increase in risk of DM for all patients, but not all patients have extensive microscopic disease. It is more likely that there will be no increase in risk for those with limited MDE, and to model this interaction, a predictive biomarker is required.

Imaging biomarkers describing density and texture features of the tumour and peritumour contain biological and prognostic information, which can help in understanding how the tumour invades into the surrounding tissue and leads to DM (10). Simple metrics from pretreatment computed tomography (CT), such as circularity and density on the surface of the gross tumour volume (GTV), can predict MDE risk (3). This risk model predicted local-regional failure, but only for a group that received a low dose up to 1.5 cm outside the GTV. This demonstrates an interaction where imaging biomarkers can stratify patients for MDE risk, and importantly this can lead to changes in the way radiotherapy is delivered (e.g., increased margins or dose). Such interactions underpin personalised RT (11).

Few imaging biomarker studies investigate dosimetric parameters, and vice versa, and their interactions are therefore not often described (12). Disregarding such important interactions can lead to studies incorrectly claiming a lack of association (13). When investigated, however, this has typically involved the use of arbitrarily defined thresholds to split patients into *‘low vs. high risk’* or to describe *‘underdosage vs. adequate coverage’* of target volumes (3). Dichotomisation of data should be avoided in the exploratory stage, as it can cause residual confounding, lead to false positive results, or underestimate the true added value of a variable (14). Studying continuous interactions is recommended but has yet to be investigated for tumour/peritumour density and dose (11). It is currently unknown whether the tumour or peritumour contains the most prognostic information, so studies have investigated shells surrounding the GTV (10, 15, 16). In addition, there is no consensus on what location should be investigated for dose (3, 9, 17).

Data mining techniques are useful for combining spatial information into the exploratory stage of analysis with no *a priori* hypothesis. In this work, we introduce a data-mining technique (*‘Cox-per-radius’*) in which a Cox regression is performed for combinations of density and dose metric derived in annuli around an automatically segmented GTV, allowing assessment of spatial density–dose interactions whilst accounting for clinical variables. In this study, we explore this method for prediction of distant metastasis following lung SABR.

## 2 Methods

### 2.1 Clinical Data

#### 2.1.1 Data Collection

Data were collected for 273 T1–2 N0M0 NSCLC patients (confirmed histologically or suspected on radiology) who were treated with SABR for primary lung cancer during 2011–2017 at The Christie NHS Foundation Trust. Ethical approval for data collection and analysis was granted by the UK Computer Aided Theragnostics Research Database Management Committee (research ethics committee reference number: 17/NW/0060). Planning four-dimensional CT (4D-CT) scans [described in Davey et al. (18)] and 3D dose distributions were available for all patients. Patients received a dose of 54 Gy in 3 fractions, or 60 Gy in 5 or 8 fractions, with further planning details included in **Supplementary Material**, Section 1. Clinical variables available were tumour lobe location, T stage, age, sex, ECOG performance status (functional ability), ACE27 comorbidity score (presence and severity of preexisting conditions), and histological subtype. Information was also available on the number of treatment fractions and the treatment type (IMRT vs. VMAT). Clinical variables were only included if they were available for over 90% of patients.

#### 2.1.2 Patient Follow-up

Patients underwent clinical follow-up 4 to 6 weeks after treatment, every 3 months for a year, and then 6 monthly thereafter. Follow-up CT was performed at the discretion of the clinician, with an 18F-FDG positron emission tomography scan and/or biopsy for suspected recurrence. Data on treatment failure were retrospectively collected from electronic records. Distant metastasis was defined as recurrence in an uninvolved lobe, contralateral lung, or any other extra-thoracic location. Time to DM was recorded from the start of RT to the date of the first scan that showed progression. Patients were censored at the most recent follow-up in the absence of failure.

### 2.2 4D-CT Density Biomarkers

#### 2.2.1 Imaging Data

Treatment plans included a ‘motion-adapted’ GTV (iGTV) which incorporates the tumour volume and its motion across all respiratory phases. This is outlined on the maximum intensity projection (MIP) and edited on individual respiratory phases to cover the tumour motion observed on 4D-CT. The slice thickness for contouring was 3 mm, and a fluorodeoxyglucose (FDG) position emission tomography (PET)-CT was available for all patients and considered as per ACROP-ESTRO recommendations (19). The inclusion of tumour spiculations was considered case by case dependent on the morphological appearance, size of spicule, and PET-CT information. In routine practice, contours are reviewed at a peer review meeting and adjusted if required.

**Figure 1:**
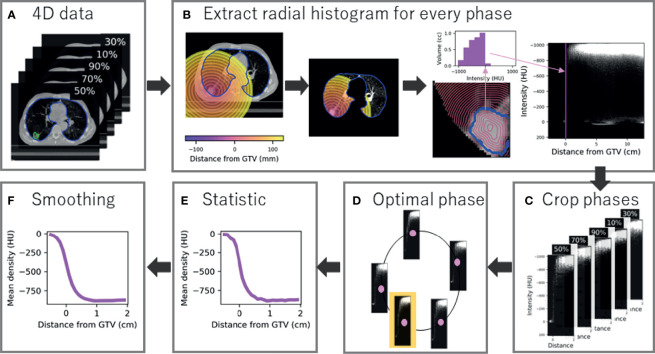
Radial histogram methodology for 4D-CT data. **(A)** A GTV and lung contour is available and optimised to each phase of the 4D-CT data (five phases for example). **(B)** A radial histogram is extracted from every phase. First a signed distance transform map is created, which is cropped to lung tissue only, and finally an intensity histogram is extracted for every 1-mm annulus and mapped onto a 2D cross histogram. **(C)** Radial histograms for every phase are cropped to sample tumour and peritumour only. **(D)** The optimal phase histogram is selected by maximising the structural similarity matrix between neighbour phases. **(E)** Summary statistics are extracted at every mm with mean density shown for example. **(F)** Gaussian smoothing is applied to the curve to limit the influence of noise.

An in-house validated technique was used to segment the GTV on all phases retrospectively (20). Briefly, local rigid registration was used to estimate the translation required to match the tumour position on each phase to a reference phase (50%). From the estimated motion, the GTV was derived and transferred to all 4D phases. All GTVs were visualised by a single observer and approved as they outlined the visible macroscopic tumour (20). A by-product of GTV generation was two additional clinical variables: GTV volume and the amplitude of tumour motion. The lung contour from the average scan was also adapted to each phase using morphological operations and thresholding (18). Example data are shown in **Figure 1A**.

#### 2.2.2 Creation of Radial Histograms

A radial histogram framework was constructed to obtain a measure of density at radial distance from the generated GTV for all patients. Firstly, a signed distance transform was applied which assigns each voxel a value representing the distance from that voxel to the nearest voxel at the border of the GTV, forming a distance scale of 1-mm annuli that are negative inside the border. Both the image and transform map were then cropped so only the lungs are considered to avoid density being linked to location. A 2D cross-histogram (*radial histogram*) was created for every 4D phase where each pixel value is the volume of the given 1-mm shell (horizontal axis) that is occupied by a particular density (vertical axis), shown in **Figure 1B**. Every histogram was cropped between -0.5 and 2 cm to select tumour and peritumour only, therefore sampling 25 annuli (**Figure 1C**). This region was selected, as we believe predictive information about MDE presence (that could be beyond 2 cm and invade surrounding structures) will be captured by tumour characteristics and peritumour invasion that describes likelihood of spread, rather than features at the exact MDE location (as this cannot be detected on CT).

#### 2.2.3 Optimal 4D Phase Selection

To determine which 4D phase to use for analysis, we found the *‘optimal phase’* individually for each patient (**Figure 1D**). The optimal phase is the most stable compared to neighbour phases and improved model performance in a preceding radiomics study (18). Although often assumed to be the case, the optimal phase is not necessarily end-exhale due to 4D-CT artefacts and the impact of motion that is not related to the respiratory cycle (e.g., cardiac motion). Adapted from the previous study, each cropped radial histogram was compared to its neighbour by the mean structural similarity index (SSIM), which is normalised to the range 0 to 1, where 1 is a perfect match. For a given phase, *Ph*, the sum of the SSIM compared to neighbour phases is


(1) $$\begin{array}{c} SSIM_{total}=SSIM\left(Ph,\;Ph+1\right)+SSIM\left(Ph,Ph-1\right) \end{array}$$

which is maximised for the optimal phase. The optimal radial histogram was selected for each patient and the corresponding phase compared to previous work.

#### 2.2.4 Summary Statistics

For each patient, first-order statistics summarised each 1-mm annulus to produce 1D curves of density feature over distance (**Figure 1E**). Overall, three summary curves were extracted per patient: mean, standard deviation (SD), and 90th percentile. Gaussian smoothing was applied to each curve as the annuli thickness was less than the slice thickness (3 mm). The standard deviation of the smoothing function, σ, was set to 1.5 mm which is comparative to the reported interobserver variation of contouring SABR cases (**Figure 1F**) (21).

### 2.3 Radial Dose Patterns

#### 2.3.1 Dose Radial Histogram

**Figure 2:**
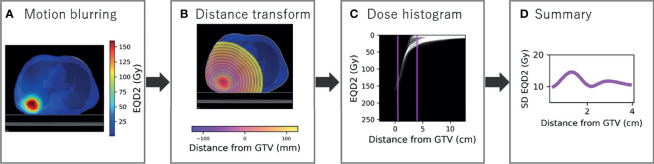
Radial histogram methodology for dose distribution. **(A)** The dose distribution is blurred for respiratory motion using 50% as reference and converted to a biologically equivalent dose in 2 Gy per fraction (EQD2). **(B)** The signed distance transform map is created to sample dose at distance from the GTV and cropped inside the body. **(C)** Radial histogram which is cropped to 0.5 to 4 cm. **(D)** Standard deviation of EQD2 at distance from the GTV summarised from the cropped radial histogram.

Similar curves were produced to describe dose over distance. Firstly, the dose distribution was blurred according to the respiratory motion to estimate the dose planned to the tumour and converted to EQD2 using α/β = 10 (**Figure 2A**). The signed distance transform map was created based on the GTV in the reference phase and cropped inside the body as MDE can be present outside the lung tissue (**Figure 2B**). The GTV was used as opposed to the PTV to link distances to spread of MDE. Dose radial histograms were generated and cropped to 0.5 to 4 cm, purposefully offset from the CT region as we are interested in the dose to the actual location microscopic disease is present (**Figure 2C**). In total, 35 dose annuli were considered.

#### 2.3.2 Summary Statistics

Standard deviation EQD2 was extracted in each annulus and the same smoothing applied as to the density data for consistency (**Figure 2D**). The generalised mean was also calculated as described by the formula


(2) $$Generalised\;mean={\left(\sum_{I=1}^{N}v_{i}D_{i}^{a}\right)}^{\frac{1}{a}}$$

where *v_i_* is a weight factor described by the fraction of annulus volume containing a dose of *D_i_*, for all potential dose values (*i* = 1,., *N*) (22–24). The parameter *a* tends to the maximum dose for a → ∞ and minimum dose for *a* → –∞. A value of -5 has been recommended for radiosensitive tumours (25), and lymph node metastases (26), but a higher value is likely appropriate for MDE due to the shallow dose–response curve and small likelihood of tumour deposits in each annulus (27). Integer values between -1 and -5 were tested to select an appropriate value. The third metric calculated was the fraction of volume in each 1-mm rim that receives EQD2 of less than 30 Gy, as an arbitrary dose threshold for inadequate MDE treatment (28).

#### 2.3.3 Comparison of Treatment Protocol

The mean dose curve was extracted for exploratory analysis. The mean and SD dose curves were visualised for patients grouped on number of fractions and treatment technique (IMRT vs. VMAT) to determine whether analysis should be limited to a particular subgroup.

### 2.4 Cox per Radius

#### 2.4.1 Interaction Map

A baseline clinical model was built to predict DM using remaining clinical variables (*i* = 4,..., *N*), and the concordance index (C-index) was extracted. For each combination of density feature and dose metric (nine in total), there were 35 annuli to extract the dose value, and 25 annuli to extract the density value, leading to 875 dose–density combinations for each metric combination. For each combination, an interaction Cox model was built containing the clinical variables, the density feature, the dose metric, and an interaction term (density*dose). The model is formulated as follows:


(3) $$\begin{array}{c} \ln\left(h(t\right))=\;\ln\left(h_{0}(t\right))\;+\;b_{1}Dose\;+\;b_{2}Density\;+\;b_{3}\left(\text{Dose}*\text{Density}\right)+\;\sum_{i=4}^{n}b_{i}*Variable_{i}\; \end{array}$$

where *h*(*t*) is the hazard function determined by the covariates included, *h*_0_(*t*) is the baseline hazard, and the coefficients (*b*_1_, *b*_2_, *b*_3_,…) describe the size and direction of effect. A separate model was also produced with the interaction term not included.

**Figure 3:**
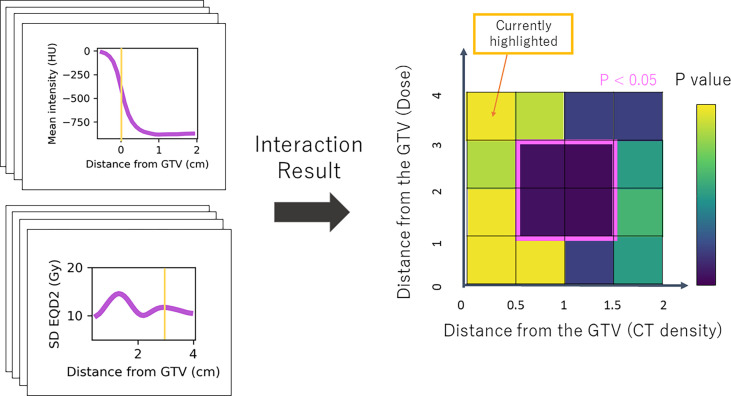
Simplified schematic of the Cox-per-radius method. The value of density feature and dose parameter is extracted at combinations of distance from the GTV for every patient. These values are used to build the Cox models and assess the significance of the interaction term. The p-value of the interaction term from a likelihood-ratio test is included in the heatmap at the distance combination. The example highlighted shows mean density at 0 cm from the GTV and standard deviation dose at 3 cm. The corresponding p-value for this interaction is included at (0,3) on the map. An example significant region demonstrates an interaction between density 0.5 and 1.5 cm from the GTV and dose 1–3 cm.

As we were interested in the locations at which density has a modifying influence on dose (or vice versa), we analysed the significance of the interaction term by performing a likelihood ratio (LR) test for the models with and without the interaction term. The test statistic of the difference in log-likelihood between the two models has a chi-squared distribution from which a p-value is obtained (29). The resulting p-value for each combination was included in a 2D heatmap at the distance of the annulus in which density was extracted on the horizontal axis, and at the distance at which the dose was extracted on the vertical axis (**Figure 3**). Statistically significant regions (p < 0.05) were selected. This was repeated for all nine combinations of density feature and dose metric. To limit the influence of multiple testing, considering each combination map independently, only regions that showed significance across several annuli were taken forward as described in the following.

#### 2.4.2 Post-processing

Post-processing was implemented as a form of multiple testing correction as there are many correlated variables, so tests are not independent (30, 31). Firstly, connected regions on the significance map were detected and each was scanned to remove rows or columns of less than 3-mm thickness. The average height and width of the processed regions were used to produce a box with the same centre as the region. As shown in **Figure 3**, the x-coordinates of the box define the radial area for density which has a significant interaction with dose in the region defined by the y-coordinates. If averaging reduced the box height or width below 3 mm, it was ignored in further analysis.

The mean values for the density feature and dose parameter inside the defined regions were calculated and assessed for variance across all patients. Regions were not considered if there was near zero variance—as this does not represent a meaningful parameter. Near-zero variance was defined as less than 10% unique values, and a frequency ratio of the most common to the second most common value greater than 19% [default in the *caret* package (32)] assessed at the arbitrary 0.1-Gy, 1%, and 1-HU difference level.

#### 2.4.3 Model Building

For each remaining region, the mean density feature and dosimetric parameter value in the defined radial areas were taken forward for model building. Each positive variable was tested for skewness, and log transformation was applied if it had the impact of reducing a large skew [ ± 3 (33)]. The overall interaction models (analogous to Equation 3) were developed for each defined region built across 500 bootstrap resamples and fit to the original data to obtain median and 95% confidence intervals (CI) of the C-index. An LR test was performed on the interaction term in each case, and the number of times the interaction significantly improves the model was recorded. Distribution of the interaction coefficient across resamples was also investigated to ensure stability (34). A coefficient was considered stable if the lower and upper bounds of the 95% CI in the bootstrap distribution were the same sign. Regions were excluded if the interaction term was unstable or if the inclusion of the dose and density did not improve the C-index.

### 2.5 Model Interpretation

Coefficients and p-values were reported for complete models with and without the interaction term, where a significant interaction suggests that density modifies the effect of dose (**Supplementary Material**, Section 2). For reporting of coefficients only, the density feature and dose metric in the relevant regions were scaled to mean zero and unit variance. Typically, hazard ratios (HR) are used to interpret the size and direction of effect, but a single value cannot be used in the presence of an interaction. The hazard ratio for different values of density compared to the mean value is described by


(4) $$\begin{array}{c} ln\left(HR\right)=\ln\left(\;\frac{h\left(t,\;Density_{i}\right)\;–h_{0}\left(t\right)}{h\left(t,Density_{mean}\right)–h_{0}\left(t\right)}\right)=\ln\left[h\left(t,\;Density_{i}\right)\right]-\ln[h\left(t,\;Density_{mean}\right)](4) \end{array}$$

where *i* incorporates the range of values observed across the patient cohort. From Equation 3, this is analogous to


(5) $$\begin{array}{c} ln\left(HR\right)=\left(b_{2}+b_{3}{\text{Dose }}_{j}\right)\left({\text{Density}}_{i}-{\text{ Density}}_{mean}\right)\; \end{array}$$

Thus, the hazard ratio can only be interpreted for density at specific values of dose, and vice versa. To interpret the direction and size of the association between density and outcome, *contrast plots* were created with ln(*HR*) on the y-axis and different density values on the x-axis for the 10th percentile, median, and 90th percentile value of the dose parameter in the relevant region (35).

The correlation between dose in identified regions and dose to the tumour was investigated with Spearman’s rank correlation to aid interpretation. The correlation between density and dose metrics with tumour volume and motion amplitude was also investigated for potential confounding due to inaccurate GTV generation, which would be a particular problem at larger amplitudes.

### 2.6 Software

The radial histogram methodology was developed in a custom in-house Python package, designed in Python 3.6.9. Aspects such as image registration and GTV generation were implemented using in-house software [WorldMatch version 9.00 (36)]. All the statistical analysis methodology was developed using R version 4.0.2. A custom R package was built and linked to the Python radial histogram workflow using *reticulate* (37).

## 3 Results

### 3.1 Clinical Data

In total, 257 patients with T1–2 N0M0 treated between 2011 and 2017 were available for exploratory analysis with patient demographics and treatment details in **Table 1**. There were 11% to 55% missing data in categories histological subtype, performance status, comorbidity ACE score, and T-stage. T-stage was removed from analysis as it is redundant to tumour volume. The other variables were also removed as missing data will likely impact the prediction of DM more than removal, leaving patient characteristics: sex and age, and tumour characteristics: volume, lobe location, and motion amplitude for inclusion in a multivariable Cox model. The median follow-up was 18 months (95% CI 15–20 months) and 44 patients (17%) had DM.

**Table 1:** Patient demographics and treatment details for 257 stage I and II patients.

Characteristic	Summary^1^	N (%)
**Treatment delivery**		257 (100%)
IMRT	171 (67%)	
VMAT	86 (33%)	
**No. of fractions**		257 (100%)
3	13 (5.1%)	
5	199 (77%)	
8	45 (18%)	
**T stage**		229 (89%)
T1	152 (66%)	
T2	77 (34%)	
**Tumour motion amplitude (cm)**	0.56 (0–3.43)	257 (100%)
**Tumour lobe location**		251 (98%)
Lower	84 (33%)	
Upper	167 (67%)	
**ACE 27 comorbidity score**		193 (75%)
None (0)	7 (3.6%)	
Mild (1)	46 (24%)	
Moderate (2)	69 (36%)	
Severe (3)	71 (37%)	
**ECOG performance status**		224 (87%)
0	3 (1.3%)	
1	77 (34%)	
2	117 (52%)	
3	27 (12%)	
**Age**	76 (45 - 93)	257 (100%)
**Sex**		257 (100%)
Female	125 (49%)	
Male	132 (51%)	
**GTV volume (cc)**	4.0 (0.3–33.8)	257 (100%)
**Histological subtype**		257 (100%)
Adenocarcinoma, NOS	47 (18%)	
Carcinoma, NOS	18 (7.0%)	
Squamous cell carcinoma	42 (16%)	
Radiological diagnosis	141 (55%)	
Other	9 (3.5%)	

^1^Statistics presented: n (%); median (range).

ECOG, Eastern Cooperative Oncology Group; ACE, Adult Comorbidity Evaluation 27.

N is the total number of patients; n is the number in each category. Tumour volume is the generated GTV volume on the 50% phase.

### 3.2 Density Features and Dose Metrics

Density information was available for all patients above -0.1 cm but only for 67% of patients at -0.5 cm, as the shortest axis of the tumour is below 1 cm so a full annulus could not be calculated 5 mm inside the tumour. For each annulus, models are built on data with samples available. The same optimal phase was selected compared to previous work for 41% of patients, with the same selection more likely in patients with larger differences between phases (**Supplementary Material**, Section 3). On visual inspection, the stability of the peritumoural region in the optimal phase was confirmed, and the difference in selection was likely due to different regions investigated for stability. The optimal radial histogram was used to extract curves of density features (mean, SD, 90th percentile) outside the GTV.

**Figure 4:**
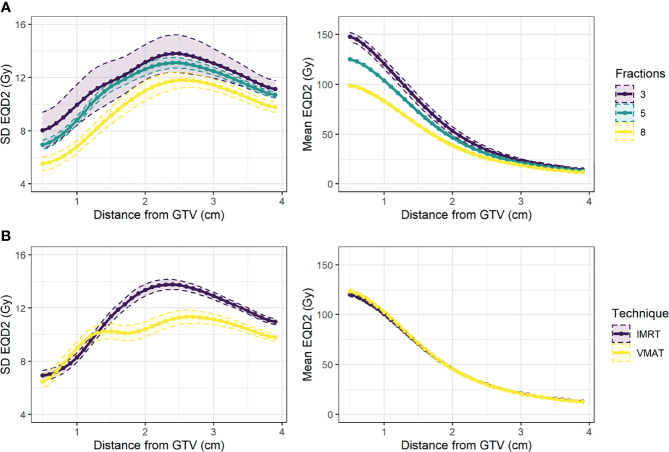
Mean and 95% confidence interval across patients for standard deviation of EQD2 (left) and mean EQD2 (right) at distance from the GTV for patients treated **(A)** on different fractionation regimes (3, 5, or 8 fractions), and **(B)** using different treatment techniques (IMRT vs. VMAT).

The dosimetric curves (SD, fraction volume <30 Gy, generalised mean) were extracted from the EQD2 dose distribution. For generalised mean, the parameter a = -3 was implemented as below this the distribution increased in skewness with small change in the average value (**Supplementary Figure 3**). The average mean and SD curves for different fractionation regimes and treatment types are shown in **Figure 4**. Most patients received 5 fractions of treatment (77%), and 67% of patients received IMRT instead of VMAT. As expected, the EQD2 dose delivered to the tumour varied for different fractionation regimes, and VMAT was associated with a more uniform distribution. There was no significant univariable association between either fractionation regime or treatment technique and DM (**Supplementary Figure 4**). For the remaining analysis, a subgroup of patients who received 5 fractions was studied to explore the influence of dose and density without any confounding due to tumour dose.

### 3.3 Clinical Model

A complete case analysis was performed on all clinical variables included, and 195 patients remained for analysis of which 36 had DM (**Supplementary Figure 5**). A baseline clinical model was built for DM with C-index = 0.64 and no significant predictors (**Supplementary Table 3**).

### 3.4 Cox per Radius

Nine *‘Cox-per-radius’* maps were produced for each density*dose interaction with at least one region identified as significant in each map and sixteen regions in total. The significance maps for all cases before and after post-processing are shown in **Supplementary Material**, Section 8. Post-processing removed a range of 3% to 56% of significant pixels across all maps. After extracting the radial distance for density and dose, four regions were below the size threshold. In addition, four regions had near-zero variance as the volume receiving below 30 Gy for the area selected (0.6 to 1.5 cm) was 0 for most patients. Three regions had interaction terms with unstable coefficients (**Supplementary Material**, Section 9). Overall, five regions remained, four of which improved the C-index of the clinical model.

The remaining models are included in **Table 2**, with the range of density and dose values reported in **Supplementary Table 6**. Two models found that the SD of density inside the tumour (1 and 2) has a relationship with DM which is modified by dose variables in different locations. The model performance was significantly improved by the inclusion of the interaction term for over 70% of resamples. The remaining models (3 and 4) had identical interpretation as the relationship between peritumour density and DM is modified by SD dose ~3 cm from the GTV and the interaction significantly improved the model for 55% and 51%, respectively. Overall, the inclusion of dose, density, and an interaction modestly improved the prediction of distant metastasis compared to the clinical model. All regions demonstrate that the association between peritumour/tumour density and outcome is modified by dose at a spatially offset location. This could support a hypothesis that peritumour and tumour density can predict the presence of MDE at distance.

**Table 2:** The models selected after assessment of region size, variance, stability, and model performance.

Region	Density parameter	Density region	Dose parameter	Dose region	C-index median (95% CI)	Freq (%)
**1**	Standard deviation	-0.5 to -0.2	Standard deviation	1.1 to 1.9	0.66 (0.61–0.69)	76
**2**	Standard deviation	-0.5 to -0.1	Fract vol <30 Gy	3.0 to 3.9	0.65 (0.60–0.68)	71
**3**	Mean	0.1 to 0.4	Standard deviation	2.4 to 3.5	0.66 (0.61–0.70)	55
**4**	90th percentile	0.0 to 0.4	Standard deviation	2.8 to 3.5	0.65 (0.61–0.69)	51

The parameter of density and dose is recorded, along with the regions from which data are extracted. C-index reports the model performance over all bootstrap resamples. Frequency reports the percentage of times the likelihood-ratio test of improvement in model performance due to the interaction term is significant (p < 0.05) over bootstrap resamples.

**Figure 5:**
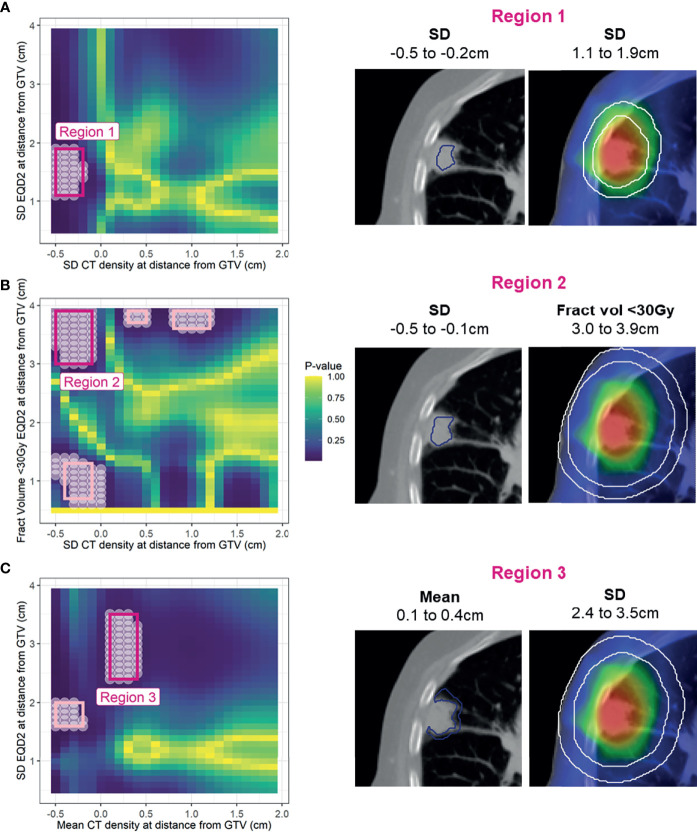
*Left:* Cox per radius significance maps of interaction between density versus dose parameters at distance from the GTV [note the difference in x- and y-axis labels for **(A)**, **(B)**, and **(C)**]. p-value represents the result of a likelihood-ratio test of improvement in model performance due to inclusion of the interaction between dose and density at each location. All significant points are shown with a white circle, and the region extracted for assessment in bootstrap is highlighted in light pink. After removal of regions that are too small, have near zero variance, have unstable coefficients, or do not improve model performance, only three remained (dark pink). *Right:* volumes defined by the selected regions on an example patient.

The spatial interpretation of the regions highlighted by the *Cox-per-radius* maps is overlaid onto an example patient CT and dose distribution for demonstration (**Figure 5**). Due to similarity in conclusion, only the first three models are shown, with Region 4 reported in **Supplementary Figure 11**. The light pink-highlighted regions on the Cox per radius map are examples of those which failed the above checks, and dark pink highlights the regions of interest.

### 3.5 Model Interpretation

The results of the multivariable models described in Section 3.4 are reported in **Table 3**, with clinical variables reported in **Supplementary Table 7**. All models have a significant interaction term, and without the interaction there is no association with outcome detected. The coefficients and p-values for density and dose in the interaction model are calculated at a reference value of zero of the interacting parameter and so have limited clinical interpretation.

**Table 3:** Coefficients (coef) and p-values for the density features and dose parameters extracted from the multivariable model for each region.

	Region 1		Region 2		Region 3	
	Coef	p value	Coef	p value	Coef	p value
***With interaction***						
Density feature in region	0.16	0.279	0.19	0.253	0.19	0.364
Dose parameter in region	-0.31	0.079	0.24	0.351	0.09	0.676
Density *dose	-0.46	**0.012**	0.47	**0.014**	0.44	**0.025**
***Without interaction***						
Density feature in region	0.15	0.334	0.13	0.435	0.20	0.303
Dose parameter in region	-0.27	0.153	0.43	0.097	0.09	0.703

The parameters have been scaled to mean zero and unit variance to aid interpretation. The interaction term is required to detect the association between density, dose, and outcome.

The values in bold represent significant p-values.

**Figure 6:**
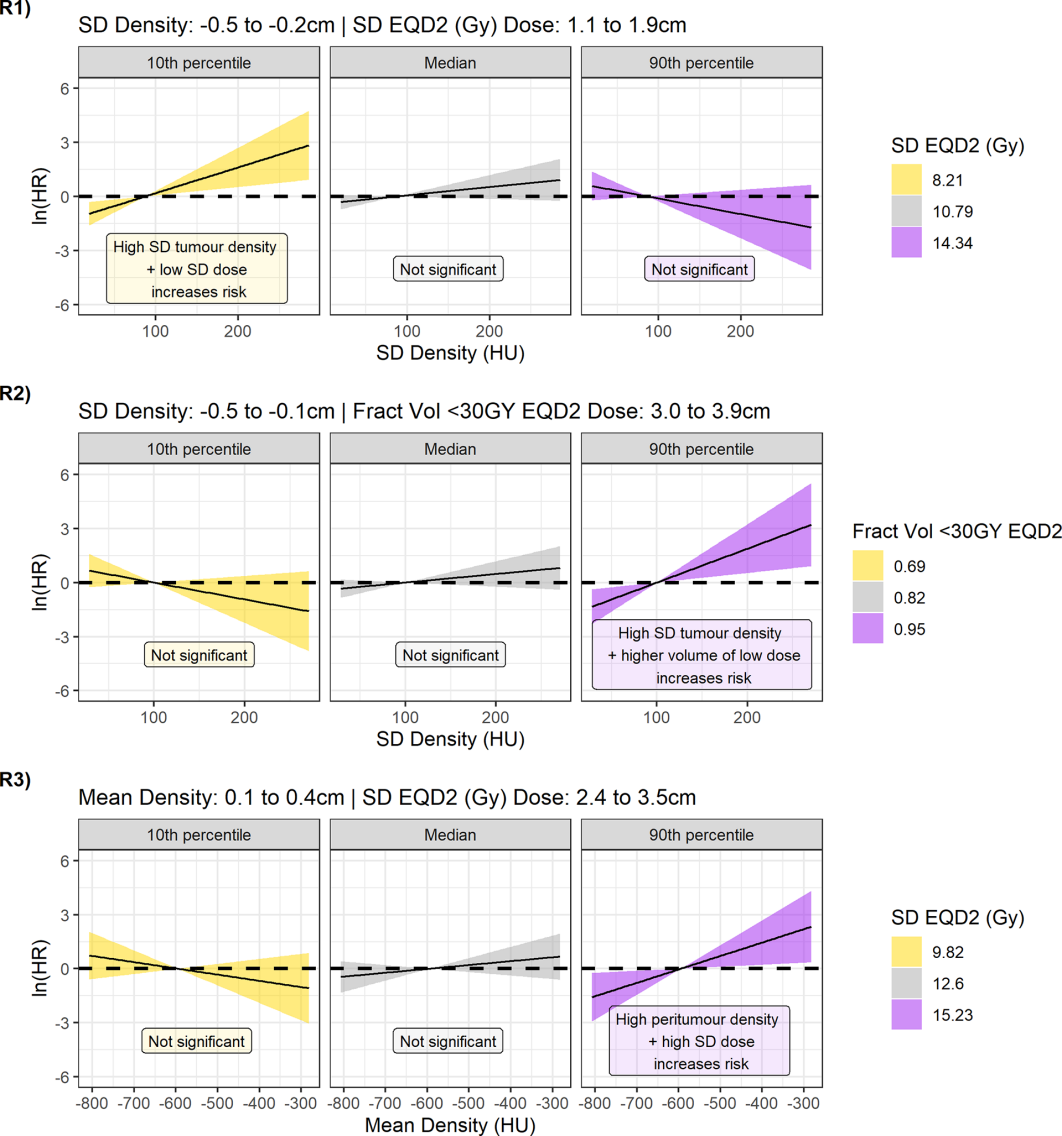
Contrast plots for each of the models built displaying the log(hazard ratio) versus density at different values of the dose parameter. From top to bottom Regions 1, 2, and 3. Significant association between density and dose is only detected at low-dose variability in the 1.1- to 1.9-cm region, and high-dose variability and greater underdosage in the 2.4- to 3.9-cm region. In these cases, higher tumour variability and higher peritumour density are associated with increased risk.

As the coefficients for density can only be interpreted at reference levels of dose, contrast plots were created for each model displaying the association between the hazard ratio and density feature for the 10th percentile, median, and 90th percentile value of the dose parameter (**Figure 6**). For regions 2 (**Figure 6**, R2) and 3 (**Figure 6**, R3), the 95% CI of the log hazard ratio includes the zero line for the 10th percentile, and median dose values. This shows that there is no association between tumour density SD/mean peritumour density and distant metastasis when there are fewer cold spots and uniform dose ~3 cm from the GTV.

An effect is observed, however, in the 90th percentile of the dose parameter. For model 2, high-density SD inside the tumour is linked to increased risk of DM if a large fraction of volume is receiving less than 30 Gy ~3 cm outside the GTV. For model 3, higher mean peritumour density is associated with worse outcome if there is non-uniform dose coverage. The lack of association for higher and more uniform doses agrees with a hypothesis that if MDE is adequately treated, biomarkers for the presence of MDE would not predict outcome.

The result of region 1 seems counterintuitive (**Figure 6**, R1), as at low dose variability (10th percentile) there is an association between tumour variability and outcome that is not observed at high variability (median, 90th percentile). Interestingly, model 1 is the only model to sample the dose variability from 1.1 to 1.9 cm outside the GTV. The contrast plots for dose for different values of density also demonstrate the reverse in risk direction dependent on the location the dose is sampled from (**Supplementary Figure 12**).

### 3.6 Confounding Investigation

The counterintuitive result of Region 1 is explained by considering the mean EQD2 dose inside each of the regions identified by the dose parameter (**Supplementary Figure 13**). In region 1, the median value is 55 Gy with range 74–92 Gy, which is significantly higher than 9 Gy (17–32) in R2 and 14 Gy (22–41) in R3. Considering the difference in dose scales, a large SD in this region would not lead to underdosage of microscopic disease.

Importantly, all dose parameters are independent of the mean GTV dose with correlation coefficients 0.26, 0.24, and -0.15, respectively. The dose is also independent of motion (-0.09, -0.26, and -0.32), as the dose has already been corrected for in the blurring of the dose distribution. The dose descriptors at larger distances somewhat correlate with tumour volume, but this is of greatest concern for region 2 where the fraction of the rim receiving a dose less than 30 Gy is measured (ρ = -0.68), compared to the models including standard deviation (0.29 for region 1 and 0.47 for region 3). The fraction of volume measurement is therefore sensitive to the increasing annulus size with larger tumours.

All density biomarkers are independent of tumour volume, 0.20, 0.17, and 0.21 for regions 1 to 3, respectively. Biomarkers representing tumour variability were completely unrelated to tumour motion (ρ < 0.01), but a higher peritumour means density weakly links to a higher motion amplitude (ρ = 0.42)—which could be an indication of under-contouring of the iGTV for large moving tumours.

## 4 Discussion

In this study, we have developed a novel methodology to explore spatial interactions of density and dose, using radial data mining and no *a priori* assumption on the most important region for extracting imaging biomarkers or dose data. This methodology is coined *‘Cox-per-radius’* and can be seen as complementary to the *‘Cox-per-voxel’* method (38). However, because the annuli are centred on the GTV, this method is applicable for tumours with variable position. This work, for the first time, has demonstrated the importance of considering how CT imaging biomarkers interact with dose to predict distant metastasis. We found that high tumour density variability and high peritumour density are associated with metastasis but only for patients who receive low and non-uniform doses ~3 cm from the GTV. Overall, the results support the hypothesis that density biomarkers predict MDE risk, and outcome is ultimately controlled by the amount of *incidental* dose received at a spatially offset location. Such interactions could inform increased margins to ensure the dose coverage for patients identified as high-risk with potential to improve patient outcome whilst minimising toxicity for low-risk patients.

The biomarkers identified are in agreement with the high surface density, high tumour density, and more complex tumour shape that has so far been linked to MDE risk (3, 5). Surface density and shape features have so far predicted local-regional failure for patients with a lower dose up to 1.5 cm from the GTV but failed to predict distant metastasis (3). The interaction between dose and density at a greater distance (~3 cm) to predict DM is described for the first time in our study. However, the dose location is similar to that observed by Diamant et al. where a lower dose ~3 cm from the PTV was found to predict DM independent of prescription dose (9). The confirmation that an *incidental* dose over 3 cm outside the GTV is independent of tumour dose suggests that studies investigating prescription dose should also consider dose outside the planned treatment area (17). The importance of considering multiple locations was further identified by the counterintuitive results displayed for Region 1. The interpretation of Region 1 is unclear, but it is located close to the edge of the PTV and the mean dose to this area is over 50 Gy for all patients, so the standard deviation of the dose may relate to conformality rather than underdosage. Further work is needed to determine its clinical relevance.

The idea of combining clinical, imaging, and treatment data to inform prediction is not new with pipelines developed to incorporate all aspects into model-building (12). However, each factor is usually assumed to contribute the same increase in risk across all patients, so interactions are ignored. If the interaction term was removed in this analysis, there was no association between density features and dosimetric parameters with outcome. However, this contrasts with the work of Diamant et al. where there was an increased risk of metastasis for lower dose across all patients (9). Further investigation is required to determine how the interaction validates in different cohorts. Overall, this study suggests that considering imaging or dose alone may underpower a study when there is biological rationale supporting that an interaction could be present (13). The link between microscopic disease and incidental dose is an example of a biological interaction, which could be relevant beyond lung cancer, for example in prostate cancer where a lower incidental dose outside the prostate has been linked to failure (39). Other examples of interactions could include the influence of genes linked to tissue radiosensitivity (40), or patient performance status on risk of experiencing a radiotherapy-related side effect (38). Although some biomarkers may not differ spatially, the ideas presented are relevant for assessment of the dose distribution whilst controlling for a biomarker of interest. Such studies could include one-dimensional radial curves, surface maps (31), or voxel-based analysis (38).

The improvement in model performance observed in this study is modest, but it is the first time this methodology has been presented and therefore density features and dosimetric parameters were chosen to be simple and interpretable. As a balance between mean and minimum dose (3, 9), we considered the generalised mean dose, but this was not selected and is likely sensitive to the *a* parameter chosen. One of the best models included the fraction of annuli volume receiving EQD2 below 30 Gy. Although investigated as a continuous parameter, defining a dose threshold without an adequate basis may lead to similar issues to subgroup analysis, i.e., increased risk of false positives (14). The threshold implemented was based on descriptions of MDE dose–response (28) and is slightly larger than a threshold previously investigated (21 Gy) (9). In addition, the fraction of volume assessment correlates with tumour volume which cannot be ignored when interpreting results. Standard deviation was also included in final models but may not always imply underdosage as SD scales with both the minimum and maximum dose. Standard deviation was not influenced by volume at distances close to the PTV—but volume becomes more important at larger distances. Despite this concern, the interaction result is significant after correcting for volume in the multivariable model.

For imaging biomarkers, we concentrated on first-order metrics which have shown potential (3), but we did not consider tumour shape or more complex texture metrics due to increased potential of tumour volume confounding (41, 42). The reduced chance of volume confounding with first-order density metrics was confirmed in this work. Testing more imaging features and dosimetric parameters would require integration of feature selection techniques, which was deemed inappropriate due to the sample size in this study. One concern related to the sample size is the multiple tests performed with an exploratory data mining methodology. To reduce the number of false positives, we implemented post-processing based on region size, investigated the stability of coefficients over bootstrap resamples, and considered the variance of the values extracted. Post-processing techniques on region size have been implemented elsewhere as a replacement for traditional multiple testing correction methods (i.e., false discovery rate), which often assumes that tests are independent (30, 31). As we are studying changes over the distance, there will be a high level of correlation between neighbouring annuli and traditional multiple testing is likely too strict. In future, we aim to test the sensitivity of the post-processing techniques against other options such as permutation testing based on model performance statistics. Overall, with post-processing on size, we identify larger regions in comparison to surface metrics reported so far (3).

The aim of this study was to describe the *‘Cox-per-radius’* methodology for the first time, but further work is required to confirm any conclusions and test sensitivity to changes in the analysis pipeline. The imaging biomarkers reported should undergo the same level of rigorous testing as traditional radiomics (43), but smoothing over radial distance and use of the *optimal phase* may already control for the impact of motion and voxel size (15, 18). However, there are different methods available to compare cross-histograms to determine the optimal phase (44).

One potential limitation is whether the GTV under-samples the ground-truth tumour volume. In the GTV generation methodology, such under-sampling could result from an iGTV which does not cover the full motion extent of a mobile tumour. This concern is highlighted in the weak correlation between tumour motion amplitude and peritumour density, but the removal of inadequate contours on visual assessment reduces the chance of under-sampled contours being included in the study (20). If inadequate contours were included, higher density could represent tumour that was missed in the original plan, but this is not limited to our approach as even gold-standard contours do not always include high-density spiculations at the border. Before full clinical interpretation of results can be made, investigation is required on whether uncertainty in contouring (i.e., inclusion of spiculations) could influence the risk of distant failure, and hence the likelihood that density biomarkers relate to microscopic disease rather than missed macroscopic disease. A key advantage of the *Cox-per-radius* approach is that confounders can be corrected for in the exploratory analysis, but like in other data-mining studies, a full causal interpretation of results cannot be gained at this stage. For use of this methodology in clinical studies, we would recommend testing of negative controls, such as the testing on voxel-randomised CT data presented by Welch et al. (41). If the outcome is linked to density rather than the region of interest, the interaction should disappear in such a test.

As we are presenting this methodology for the first time, uncertainty remains on the biological correlation between the dose relationships identified and the development of distant metastasis. Like in other microscopic disease investigations (3, 9), we did not limit the assessment of dose to the surrounding volume to anatomical regions (such as lung tissue only). This could lead to inclusion of tissue which is not relevant for disease spread (e.g., skin, heart) in the defined annuli (see **Figure 5**), but this is dependent on tumour location. The inclusion of a link to anatomical location in this methodology would be interesting as little is known about tumour location and recurrence after SABR, as reports on disease spread to chest wall, mediastinum, bronchi, and vessels are limited to surgical cohorts (45). A quantitative assessment of location will provide more information than the tumour lobe location variable currently included in the models (46, 47). Assessing specific anatomical locations such as ‘lungs only’ is a potential option to reduce spurious correlations but will lead to different amounts of data excluded dependent on location which could introduce bias. Furthermore, data-mining techniques could provide information on less understood biological mechanisms, such as immune suppression, where the inclusion of surrounding anatomy may be important (48). In addition, it may be important to assess the molecular and histological characteristics of the tumour when providing a complete picture of metastatic potential—but this was limited by data availability.

Overall, although further work is required, we have demonstrated that spatially offset interaction models can be built including CT density biomarkers and dosimetric parameters whilst controlling for potential confounding variables. The inclusion of interactions is key to personalised radiotherapy, so that high-risk patients can be identified, and treatment changes can be implemented to improve patient outcome.

## 5 Conclusions

The interaction between imaging biomarkers and dose has been under-investigated in radiotherapy research. We have successfully developed a novel image-based data mining method to explore interactions between imaging biomarkers and dosimetric parameters at radial distance from the tumour. The methodology allows for simultaneous assessment of CT density and dose on independent distance scales. Density biomarkers interact with dose to predict distant metastasis, and associations with DM were not found in the absence of the interaction term. Higher tumour density variability and peritumour density are associated with increased risk of DM if there is a larger chance of underdosage (<30 Gy) and non-uniform dose ~3 cm from the GTV. The dose regions identified are independent of tumour dose, but dose standard deviation does not adequately describe underdosage at all distances leading to counterintuitive results within 2 cm outside of the GTV. Overall, most models support the hypothesis that density biomarkers can predict MDE risk, and adequate dose coverage outside the planned treatment area is required to reduce risk of treatment failure.

## Data Availability Statement

The data analyzed in this study are subject to the following licenses/restrictions: ethical permission was not granted for general publication of the dataset. Requests to access these datasets should be directed to Dr. Alan McWilliam, alan.mcwilliam@manchester.ac.uk.

## Ethics Statement

Our retrospective analysis of anonymised routine data was approved by institutional information governance and research ethics committee (The Christie NHS Foundation Trust and Caldicott Committee). The research was carried out according to a protocol approved by the Caldicott Committee.

## Author Contributions

AD developed the methodology, collated the data, performed the primary analysis, and wrote the manuscript. AM and MvH conceived the project idea and provided expert insight at all stages of the experiment. CF-F provided expert clinical insight to the project. All authors contributed to the article and approved the submitted version.

## Funding

This was supported by CRUK *via* the funding to the Cancer Research Manchester Centre [C147/A25254]. MvH and CF-F are supported by NIHR Manchester Biomedical Research Centre. Open-access publication fees were provided by The University of Manchester Library.

## Conflict of Interest

The authors declare that the research was conducted in the absence of any commercial or financial relationships that could be construed as a potential conflict of interest.

## Publisher’s Note

All claims expressed in this article are solely those of the authors and do not necessarily represent those of their affiliated organizations, or those of the publisher, the editors and the reviewers. Any product that may be evaluated in this article, or claim that may be made by its manufacturer, is not guaranteed or endorsed by the publisher.
