# Supplementary material for: Radial Data Mining to Identify Density–Dose Interactions That Predict Distant Failure Following SABR
Source: Front Oncol. 2022 Mar 9;12:838155. doi: 10.3389/fonc.2022.838155 (PMC8959483; doi:10.3389/fonc.2022.838155)
Supplement: Supplementary file 1 [file DataSheet_1.docx]

**Supplementary material**

# Planning protocol

All plans were extracted from the Philips Pinnacle treatment planning system. The target volume was an iGTV outlined on the maximum intensity projection and adapted on individual respiratory phases to cover tumour motion observed on 4D-CT. There was no CTV and a 5mm iGTV-PTV expansion was used. Prescription was planned to the D95 PTV, and the collapsed cone algorithm *(Type B)* was used for calculation with a 2mm deposition grid on the average intensity projection (AIP) of the 4D-CT.

# Significance testing

The p-value reported for each covariate in the Cox model is the result of a Wald test with the null hypothesis that the coefficient for that covariate is equal to zero. In Table 1 the interpretation of these in the presence of an interaction is described.

| **Null hypothesis** | **Null conclusion** | **Alternative conclusion** |
| --- | --- | --- |
| $b_{3}$= 0 | Effect of dose is independent of density  Effect of density is independent of dose | Dose modifies the effect of density  Density modifies the effect of dose |
| $b_{1}=b_{3}$= 0 | Density is not associated with DM | Density is associated with DM for some value of dose |
| $b_{2}=b_{3}$= 0 | Dose is not associated with DM | Dose is associated with DM for some value of density |
| $b_{1}=b_{2}=b_{3}$= 0 | Neither dose nor density is associated with DM | Either dose or density is associated with DM |

**Table 1.** Significance test interpretation for a dose-density interaction. Informed by and adapted from Regression Modelling Strategies by Frank Harrell Jr (Springer, 2015).

# 4D-CT phase selection


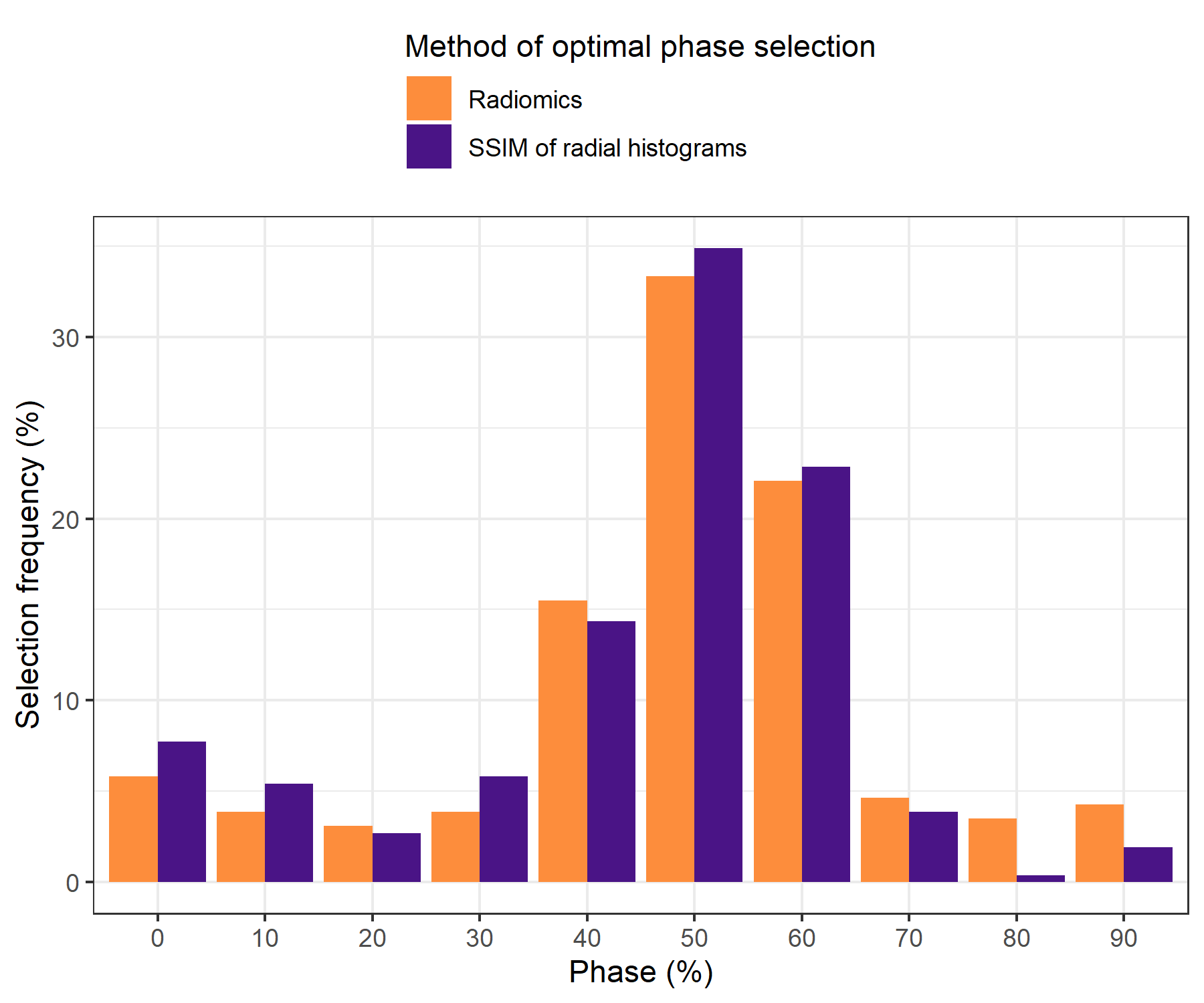
The distribution of phase selection across all patients for the new and previous published technique is demonstrated in Figure 1, with 50% as the most frequently selected in both.

**Figure 1.** Bar chart of the optimal phase selected in the radiomics approach (orange), and in the structural similarity index comparison of radial histograms (purple).


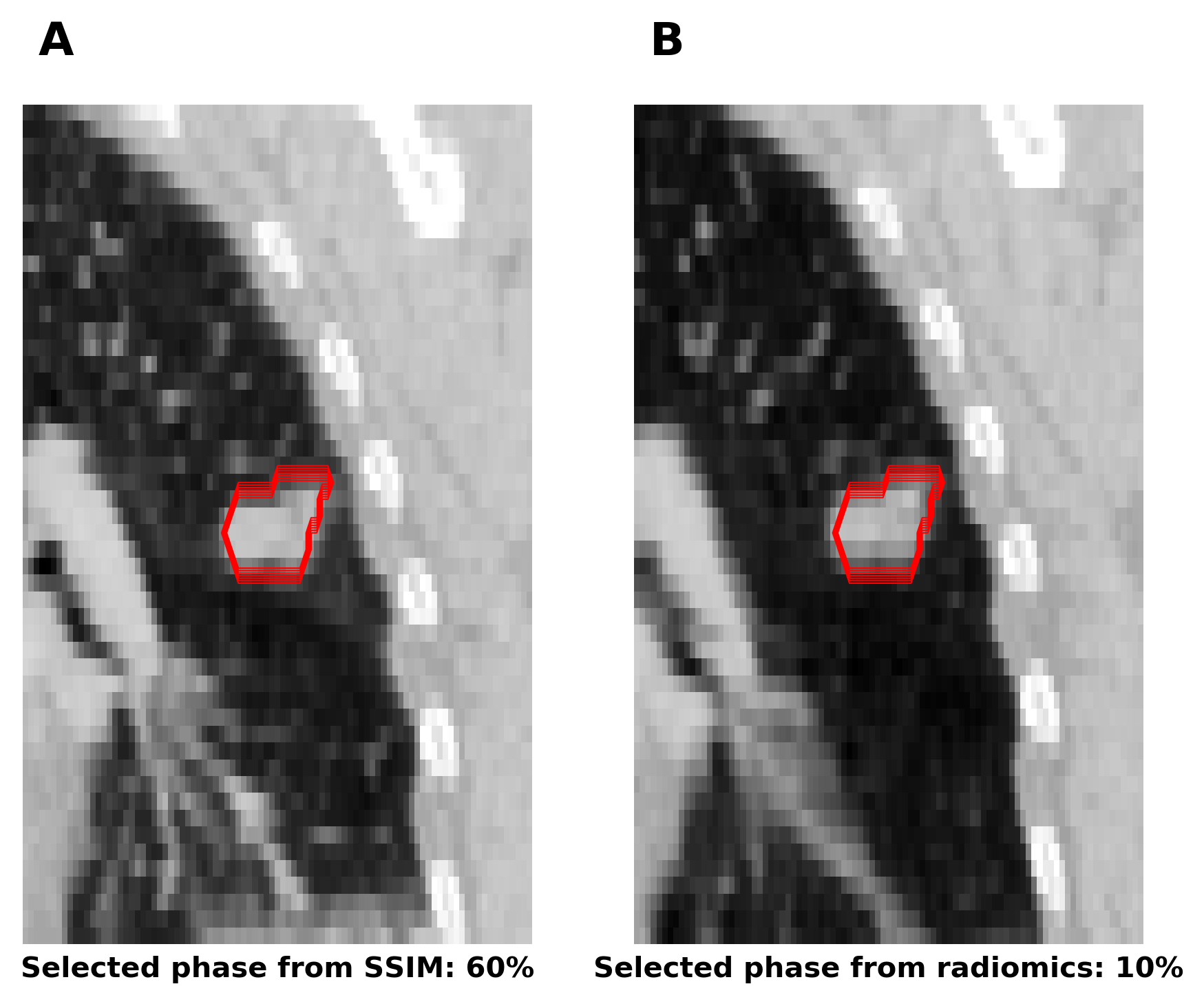
A Wilcoxon rank-sum test was performed to compare the minimum SSIM_total_ across all phases for cases where the selection matched previous technique to cases with a different phase selected. The minimum SSIM_total_ was lower in phases that did not match previous selection (p = 0.021), as more subtle differences lead to different selection where potentially many phases could be optimal. However, the difference in selection is likely due to the different location analysed (Fig. 2).

**Figure 2.** Coronal CT example of a case where the optimal phase differed in radial histogram analysis based on structural similarity matrix (left) compared to radiomics (right). The phase selected from the radial histogram has more detail in the peritumour region than the phase selected in radiomics, potentially due to difference in location as radiomics was focused on stability at the tumour and border only.

#
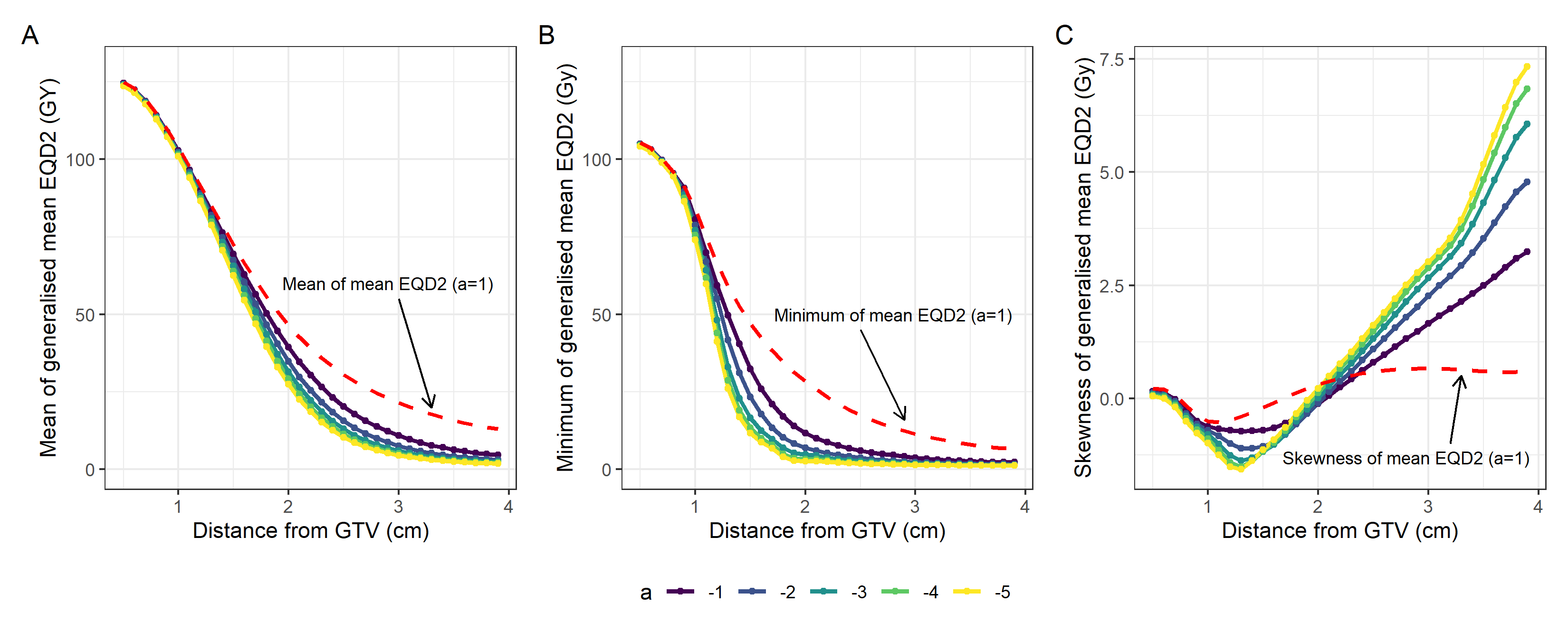
Generalised mean comparison

**Figure 3. A)** mean, **B)** minimum, and **C)** skewness over distance from the GTV for generalised mean with a = -1, -2, -3, -4, and -5. The mean (a=1) is included for comparison (red dashed line). For all parameters, the generalised mean pulls values towards the minimum, but the difference between curves reduces as the parameter increases. For values less than a = -3 the gain in reducing the distribution towards the minimum does not outweigh the disadvantage of increased skew due to outliers at distances far outside the GTV.

#
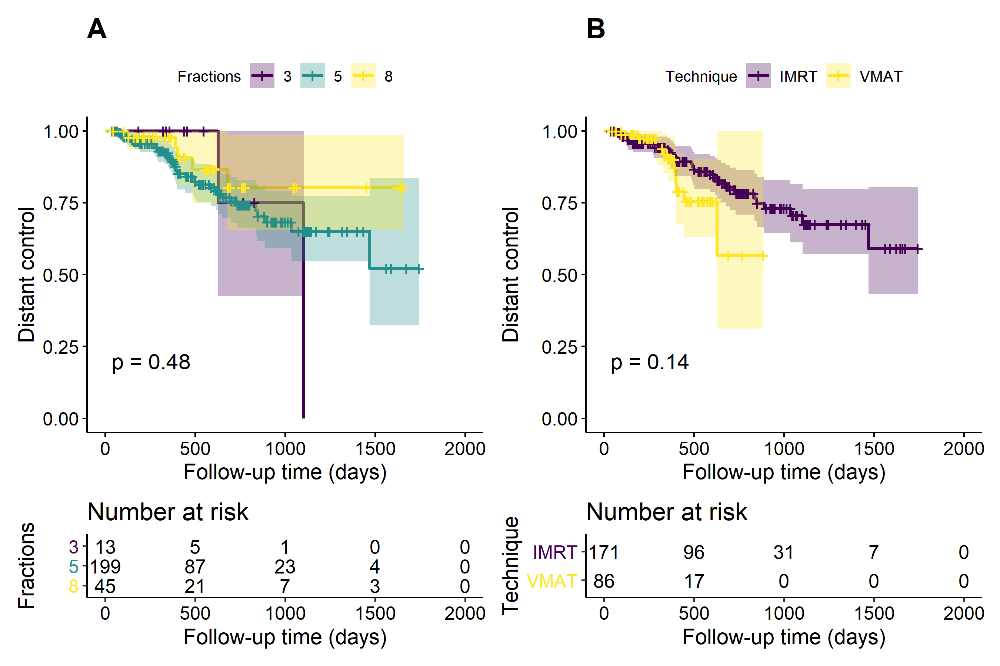
Treatment comparison

**Figure 4.** Kaplan-Meier curves for distant control show no significant difference between different fractionation regimes **(A)** or different treatment techniques **(B).**

# Patient selection


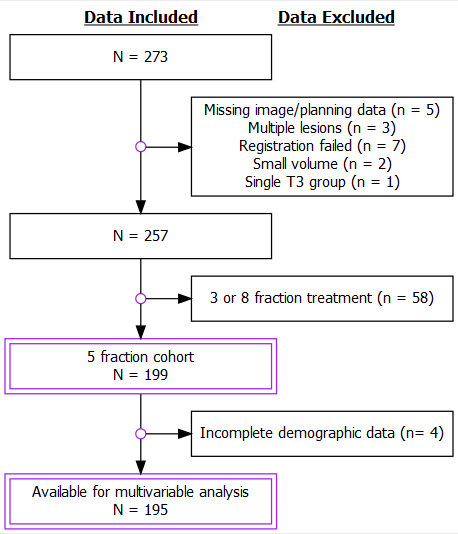


**Figure 5.** Flow diagram to demonstrate steps implemented to arrive at analysis cohort. N represents the number of patients remaining, whilst n represents those removed. Reasons for removal at step one is described in Davey et al, Phys Med Biol, 2020.

| Characteristic | Summary^1^ | N (%) |
| --- | --- | --- |
| **Tumour motion amplitude (cm)** | 0.56 (0 - 3.43) | 199 (100%) |
| **Tumour lobe location** |  | 195 (98%) |
| Lower | 69 (35%) |  |
| Upper | 126 (65%) |  |
| **Age** | 75 (45 - 93) | 199 (100%) |
| **Sex** |  | 199 (100%) |
| Female | 99 (50%) |  |
| Male | 100 (50%) |  |
| **Tumour volume [Generated GTV] (cc)** | 4.0 (0.3 - 31.0) | 199 (100%) |
| ^1^Statistics presented: n (%); Median (range) | | |

**Table 2.** Summary statistics for the variables included in the analysis for the 199 patients used.

# Clinical model

|  | HR (95% CI) | P-value |
| --- | --- | --- |
| **Tumour volume [Generated GTV] (cc)** | 1.35 (0.98-1.87) | 0.068 |
| **Tumour motion amplitude (cm)** | 0.96 (0.50-1.86) | 0.909 |
| **Tumour lobe location (Lower vs upper)** | 0.51 (0.24-1.07) | 0.075 |
| **Age** | 1.00 (0.97-1.04) | 0.878 |
| **Sex (Female vs male)** | 0.56 (0.27-1.13) | 0.106 |

**Table 3.** Clinical multivariable Cox model for baseline to predict distant metastasis. No variables are significantly associated with outcome.

#
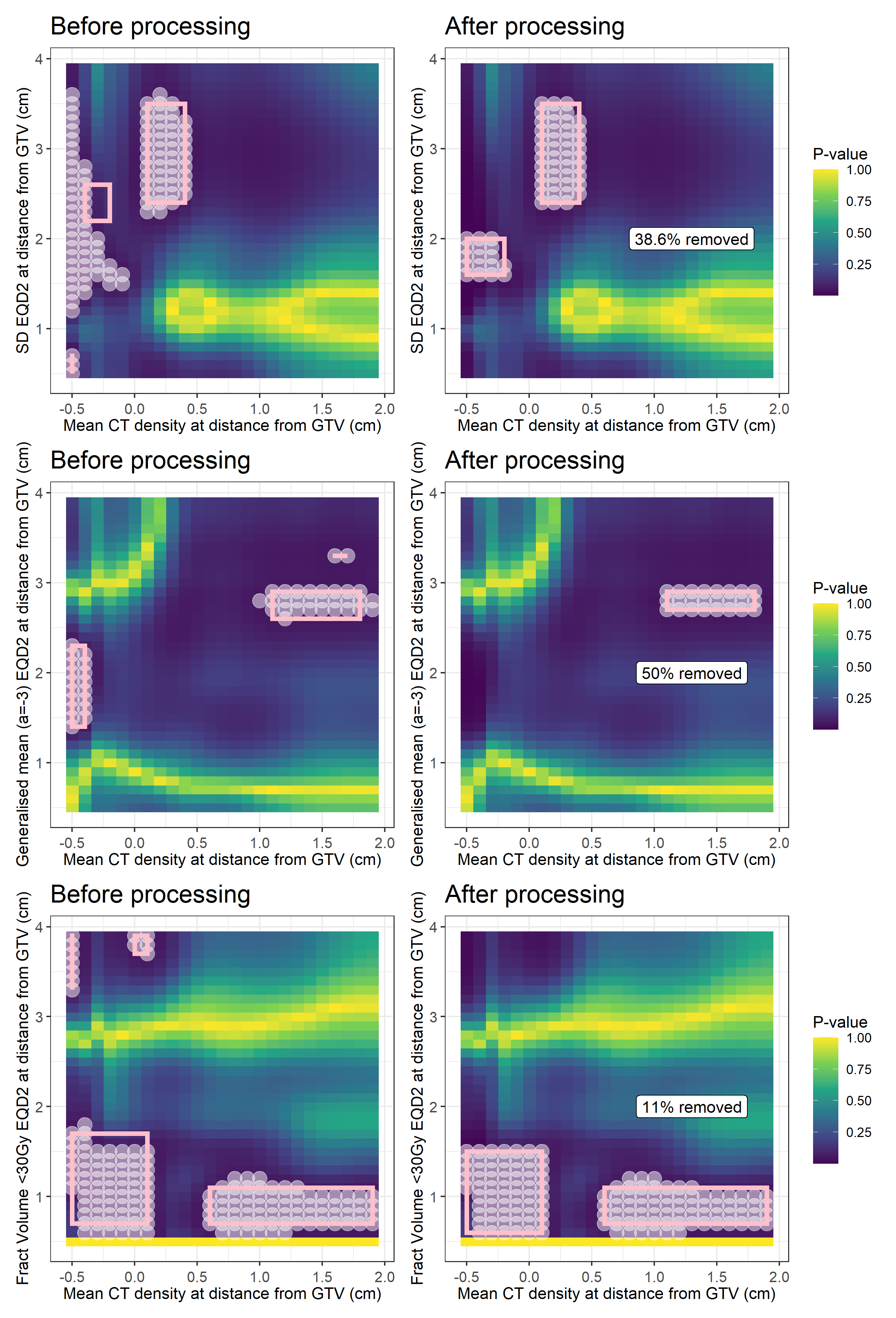
Cox-per-radius maps

**Figure 6.** Cox-per-radius significance maps for the interaction between mean CT density versus dose parameters outside the auto-generated GTV. **From top to bottom:** standard deviation, generalised mean, and fraction of annulus volume receiving less than 30Gy EQD2. The right plots show the results after post-processing with the percentage of significant points excluded.


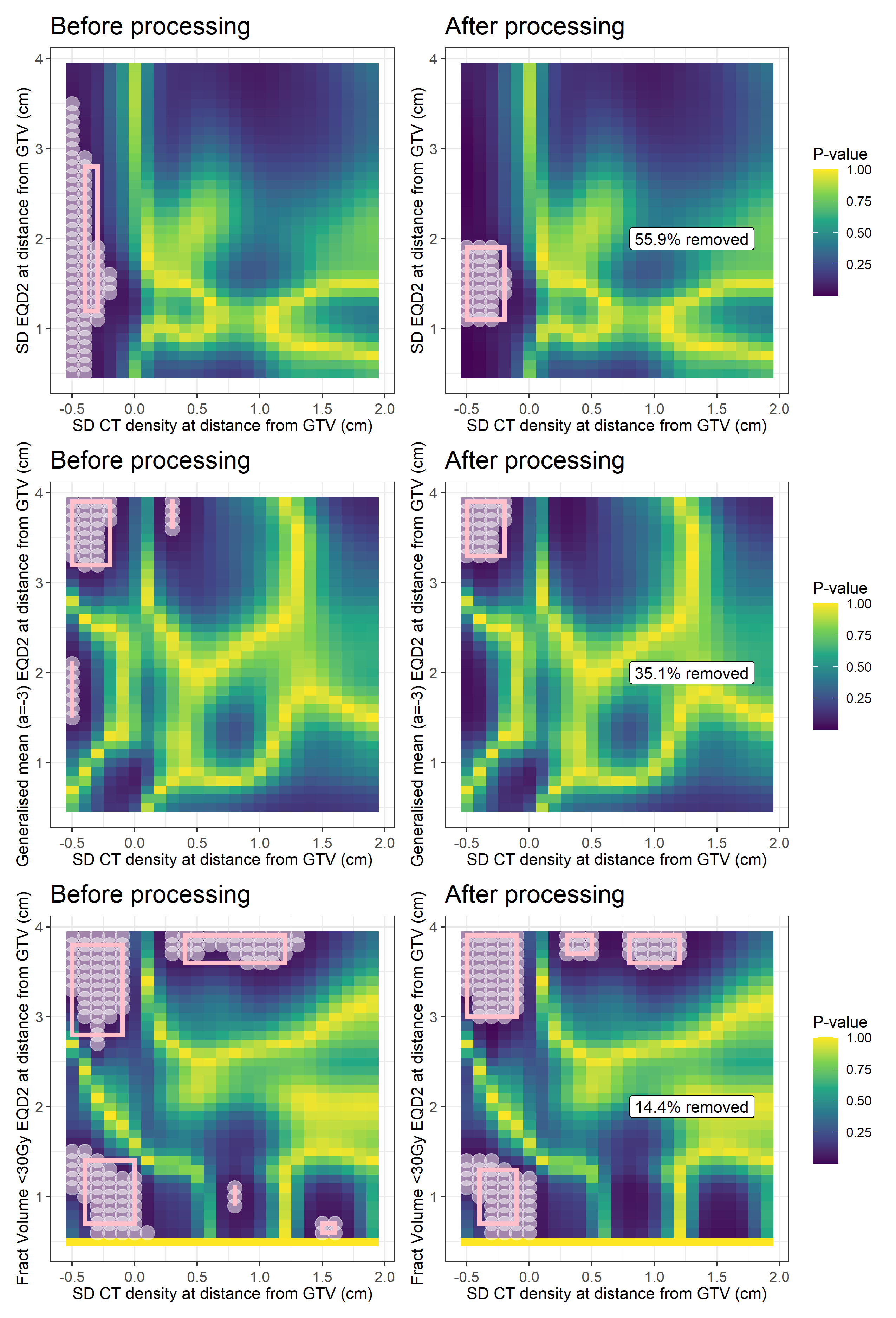


**Figure 7.** Cox-per-radius significance maps for the interaction between SD CT density versus dose parameters outside the auto-generated GTV before and after post-processing (analogous to the previous figure).


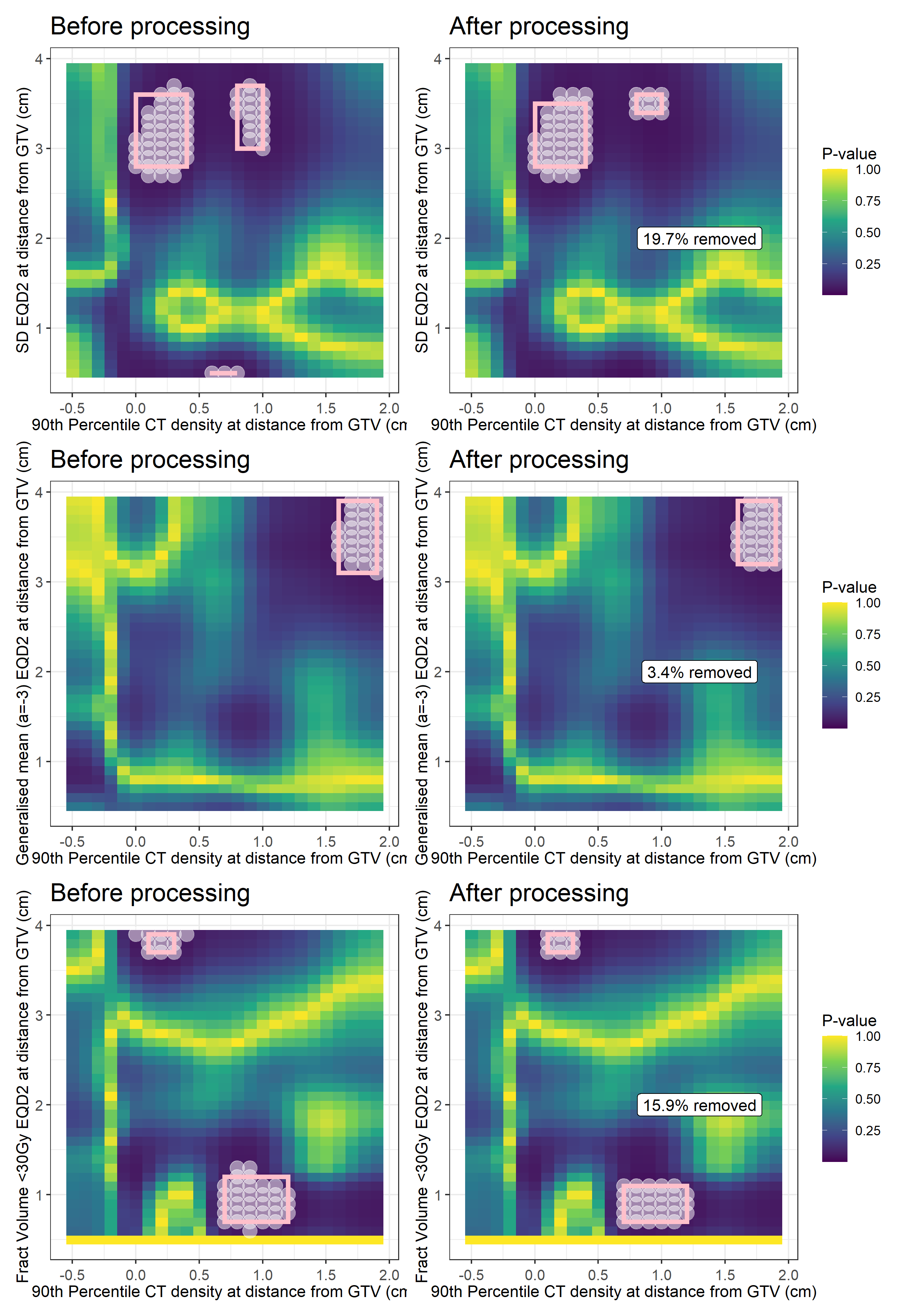


**Figure 8.** Cox-per-radius significance maps for the interaction between 90^th^ percentile CT density versus dose parameters outside the auto-generated GTV before and after post-processing (analogous to the previous figure).

# Excluded regions

| Density parameter | Dose parameter | Density region | Dose region |
| --- | --- | --- | --- |
| Standard deviation | Fract vol <30Gy | 0.3 to 0.5 | 3.7 to 3.9 |
| 90th percentile | Fract vol <30Gy | 0.1 to 0.3 | 3.7 to 3.9 |
| Mean | Generalised mean  (a=-3) | 1.1 to 1.8 | 2.7 to 2.9 |
| 90th percentile | Standard deviation | 0.8 to 1.0 | 3.4 to 3.6 |

**Table 4.** Regions that were removed on post-processing for being below the size threshold after extracting the average height and width of the highlighted region on the Cox-per-radius interaction map.

| Density parameter | Dose parameter | Density region | Dose region | Dose median (range) |
| --- | --- | --- | --- | --- |
| Mean | Fract vol <30Gy | -0.5 to 0.1 | 0.6 to 1.5 | 0 (0 - 0.03) |
| Standard deviation | Fract vol <30Gy | -0.4 to -0.1 | 0.7 to 1.3 | 0 (0 - 0.02) |
| 90th percentile | Fract vol <30Gy | 0.7 to 1.2 | 0.7 to 1.1 | 0 (0 - 0.01) |
| Mean | Fract vol <30Gy | 0.6 to 1.9 | 0.7 to 1.1 | 0 (0 - 0.01) |

**Table 5.** Regions that were removed on post-processing for being below the size threshold have near zero variance in the dosimetric parameter.


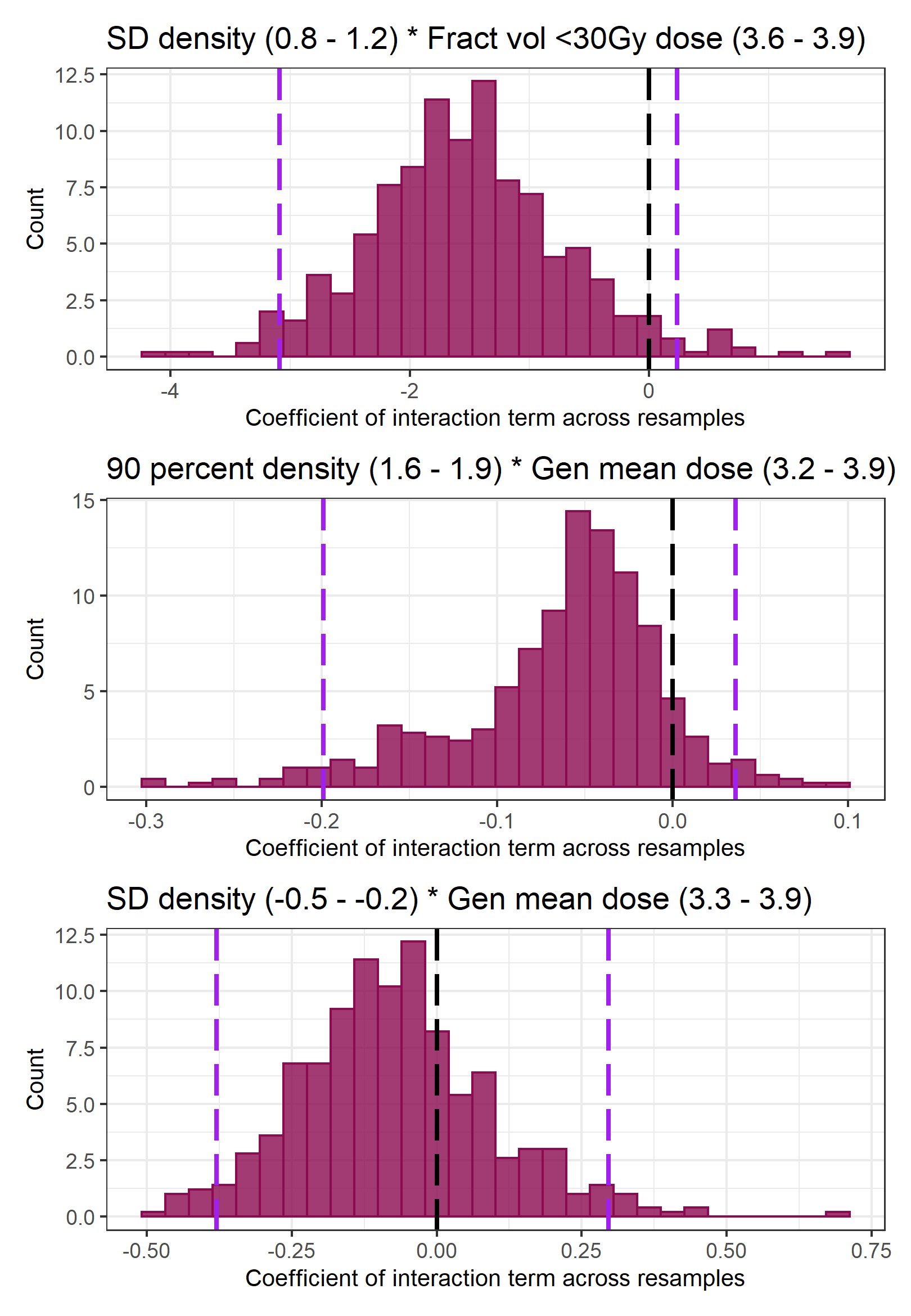


**Figure 9.** Histogram of coefficients for regions that were considered unstable in the interaction term. The purple dashed lines demonstrate the 95% confidence interval, and the black dashed line is at zero. Unstable coefficients were considered those that show both positive and negative effect. Gen: generalised, Fract vol: fraction of volume, 90 percent: 90th percentile.

# Included regions


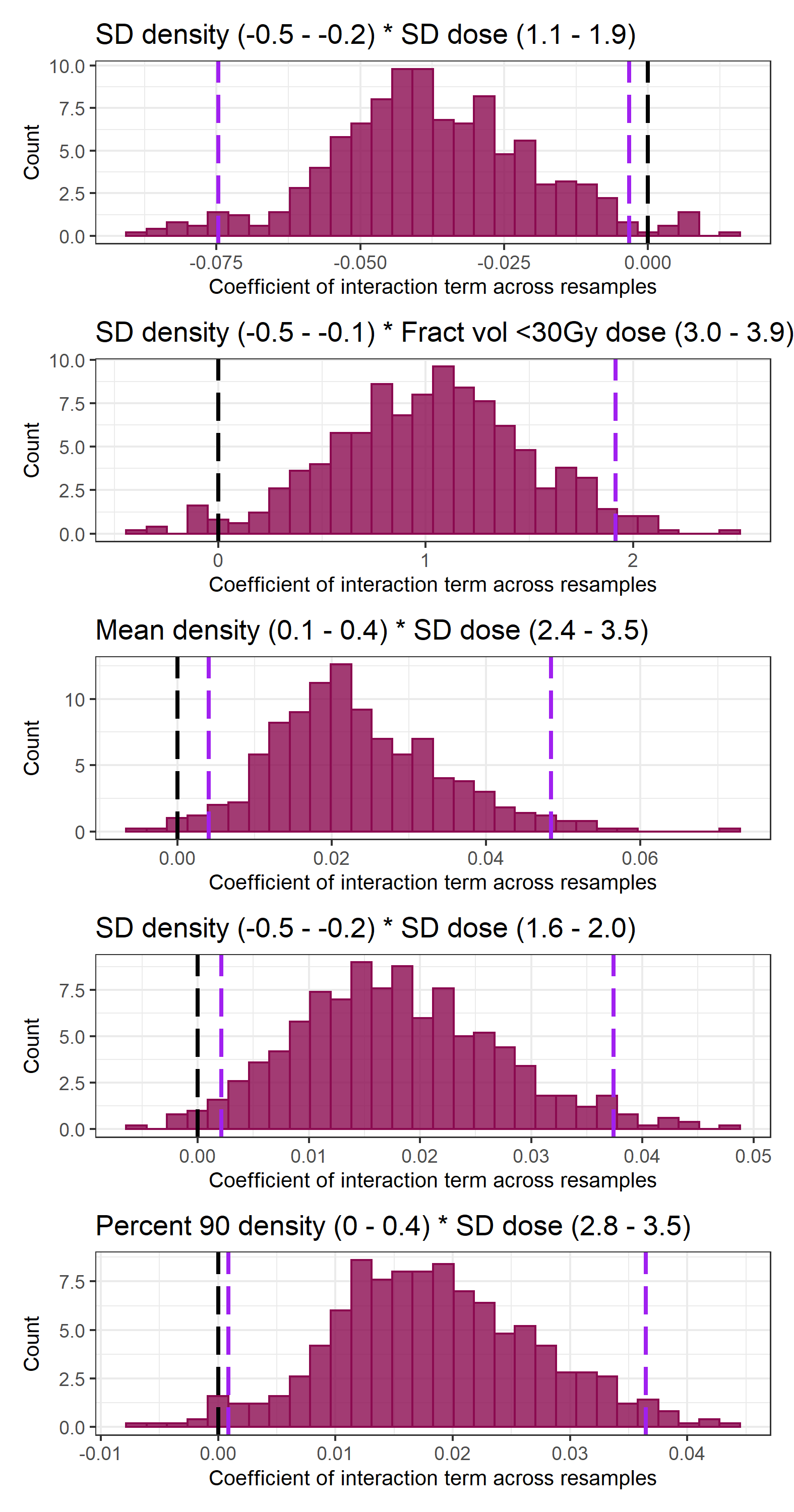


**Figure 10**. Histogram of coefficients for the five remaining regions have an interaction term with stable coefficients across bootstrap resampling.

|  | Density parameter | Dose parameter | Density region | Dose region | Density median (range) | Dose median (range) | C-index median (95% CI) |
| --- | --- | --- | --- | --- | --- | --- | --- |
| 1 | Standard deviation | Standard deviation | -0.5 to -0.2 | 1.1 to 1.9 | 75 (20 - 285) | 11 (6.4 - 20) | 0.66 (0.61 - 0.69) |
| 2 | Standard deviation | Fract vol <30Gy | -0.5 to -0.1 | 3.0 to 3.9 | 88 (29 - 271) | 0.82 (0.43 - 0.99) | 0.65 (0.6 - 0.68) |
| 3 | Mean | Standard deviation | 0.1 to 0.4 | 2.4 to 3.5 | -605 (-807 - -282) | 13 (6.3 - 18) | 0.66 (0.61 - 0.7) |
| - | Mean | Standard deviation | -0.5 to -0.2 | 1.6 to 2.0 | -48 (-399 - 34) | 12 (6 - 21) | 0.64 (0.58 - 0.67) |
| 4 | 90th percentile | Standard deviation | 0.0 to 0.4 | 2.8 to 3.5 | -340 (-632 - -50) | 12 (7.1 - 17) | 0.65 (0.61 - 0.69) |

**Table 6.** Five regions remained after post-processing on size, variance, and coefficient stability. One was excluded due to a C-index which does not improve on the clinical model performance (highlighted in orange), and the others are labelled (1-4) to match labels within the manuscript.

# Region 4


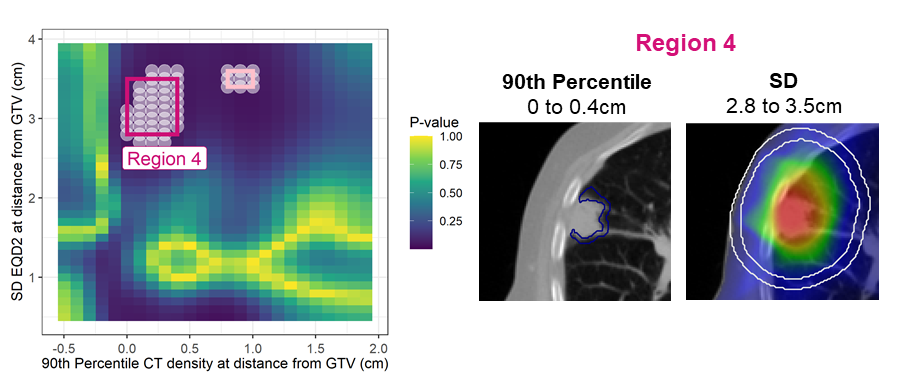


**Figure 11.** Cox-per-radius significance map (left) and associated annuli (right) for Region 4.

# Multivariable model

|  | **Region 1** | | **Region 2** | | **Region 3** | |
| --- | --- | --- | --- | --- | --- | --- |
|  | HR (95% CI) | P val | HR (95% CI) | P val | HR (95% CI) | P val |
| GTV volume (cc) | 1.46 (1.02-2.09) | **0.037** | 1.68 (1.06-2.65) | **0.028** | 1.18 (0.77-1.79) | 0.446 |
| Motion amplitude (cm) | 0.96 (0.48-1.92) | 0.904 | 1.06 (0.50-2.23) | 0.889 | 1.17 (0.60-2.31) | 0.642 |
| Lobe location (lower ref) | 0.64 (0.30-1.36) | 0.244 | 0.55 (0.25-1.19) | 0.128 | 0.63 (0.29-1.38) | 0.250 |
| Age | 1.00 (0.97-1.04) | 0.834 | 1.00 (0.96-1.04) | 0.964 | 1.00 (0.96-1.04) | 0.988 |
| Sex (female ref) | 0.51 (0.25-1.04) | 0.066 | 0.61 (0.3-1.24) | 0.169 | 0.47 (0.23-0.99) | **0.046** |
| Density feature | 1.17 (0.88-1.56) | 0.279 | 1.20 (0.88-1.65) | 0.253 | 1.21 (0.80-1.84) | 0.364 |
| Dose parameter | 0.73 (0.51-1.04) | 0.079 | 1.27 (0.77-2.10) | 0.351 | 1.10 (0.71-1.69) | 0.676 |
| Density*Dose | 0.63 (0.44-0.91) | **0.012** | 1.60 (1.10-2.33) | **0.014** | 1.56 (1.06-2.30) | **0.025** |

**Table 7.** Multivariable results table for the three models included in the manuscript.

#
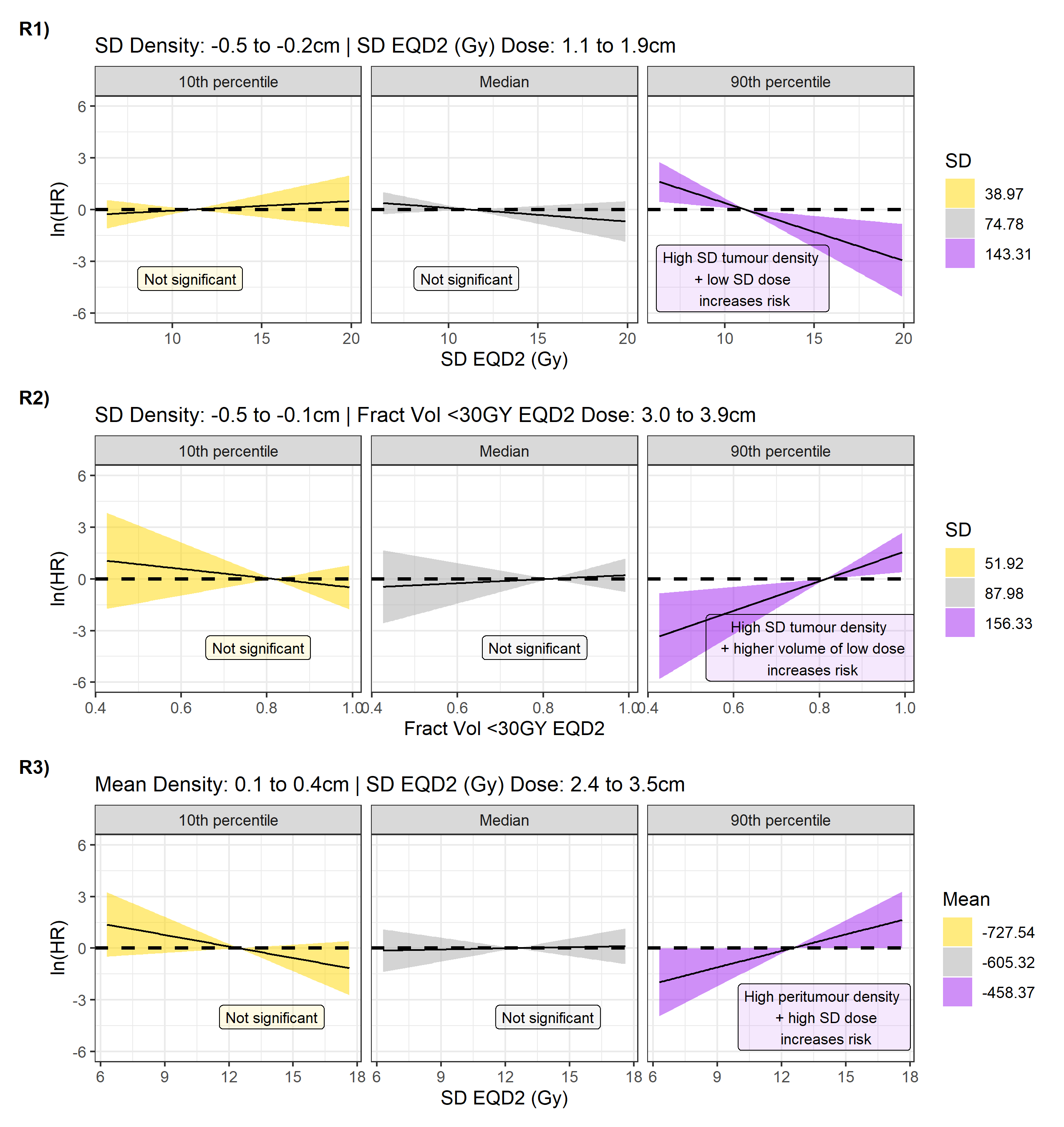
Dose interpretation

**Figure 12.** Contrast plots for each of the models displaying the log(hazard ratio) versus dose at different values of the relevant density parameter. From top to bottom: Region 1, 2 and 3.

# Region 1 interpretation


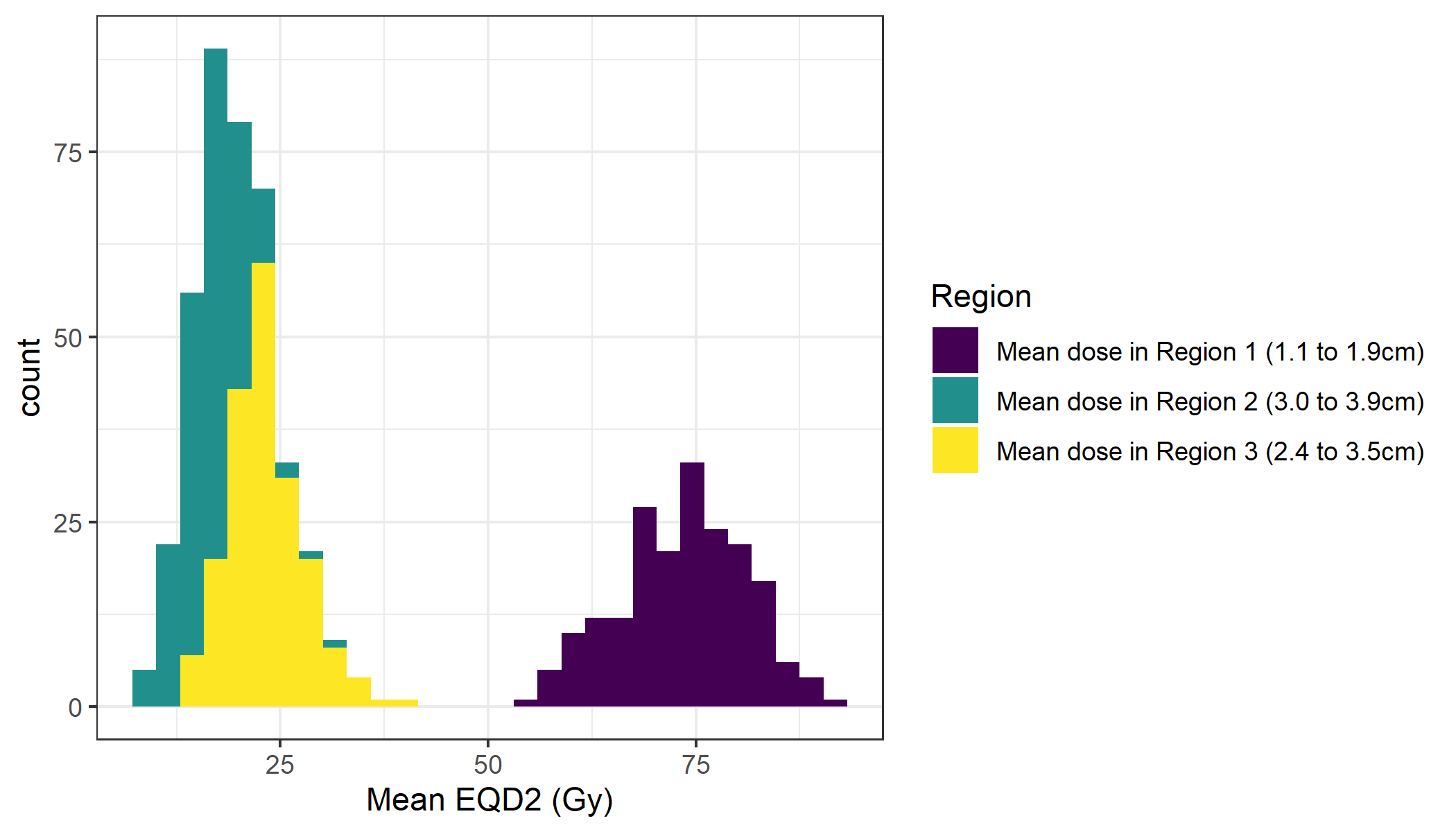


**Figure 13.** Histogram of the mean EQD2 dose in the regions identified.
